# Establishment of an industrialized micropropagation system for *Cerasus campanulata* Maxim

**DOI:** 10.3389/fpls.2026.1766373

**Published:** 2026-02-18

**Authors:** Chuwei Ding, Yueqiu He, Yongping Lv, Haojie Mou, Ying Yu, Zhilong Wang, Jianping Chen, Zhi Chen

**Affiliations:** 1 Institute of Virology and Biotechnology, Zhejiang Academy of Agricultural Sciences, Hangzhou, Zhejiang, China; 2 Ningbo City College of Vocational Technology, Ningbo, Zhejiang, China; 3 Key Laboratory of Green Plant Protection, Zhejiang Academy of Agricultural Sciences, Hangzhou, Zhejiang, China; 4 Institute of Plant Virology, Ningbo University, Ningbo, Zhejiang, China

**Keywords:** *Cerasus campanulata* Maxim., *in vitro* propagation, ISSR, medium optimization, orthogonaldesign, woody plants

## Abstract

*Cerasus campanulata* Maxim. possesses high ornamental value owing to its early flowering period, holding considerable application potential. However, research on its propagation techniques is still in the preliminary stage. To establish an efficient regeneration system for *C. campanulata*, this study used tissue-cultured seedlings as explants and employed an orthogonal experimental design to investigate the effects of key factors at different culture stages on plant regeneration. The results indicated that the suitable proliferation medium was ½MS supplemented with 0.1 mg/L NAA, 0.5 mg/L 6-BA, and 2.5 g/L banana powder. With this medium, the survival rate reached 100%, the proliferation rate was 96.67%, the proliferation coefficient exceeded 4, and the average bud height was 4.57 cm. Furthermore, the regenerated buds exhibited vigorous and healthy growth. The suitable rooting medium was ½MS, which yielded a rooting rate of over 88% and a survival rate of over 88% after greenhouse acclimatization. After 120 days of pot cultivation, the regenerated plants achieved an average plant height of 57.83 cm, an average leaf length of 15.03 cm, an average leaf width of 6.31 cm, and an average of 17 leaves per plant. Genetic stability analysis of the regenerated plantlets after three subculture cycles showed no obvious genetic variation. In conclusion, this study successfully established a practical regeneration system for *C. campanulata*, covering proliferation, rooting, acclimatization, pot cultivation, and preliminary genetic stability verification, which provides a reliable technical basis for the large-scale propagation and application of this species.

## 1 Introduction

Cherry blossoms, belonging to the genus Cerasus, are renowned for their elegant morphology, exceptional ornamental value, and cultural significance. They play a vital role in enhancing urban greening, improving environmental quality, and boosting tourism development. China has a long history of cherry cultivation and boasts the largest number of cultivated cherry trees in the world. However, research on cherry breeding has long lagged behind, resulting in relatively limited diversity among cultivated varieties, most of which are primarily introduced from Japan (Liu et al., 2018). Moreover, the exploitation and utilization of native characteristic cherry germplasm resources remain inadequate, making it difficult to meet the diversified market demands *Cerasus campanulata* Maxim., as an indigenous cherry blossom tree species in China, has an early flowering period, rich flower colors, strong adaptability, anti-pollution ability, and high ornamental value. It is more tolerant to high temperatures and has stronger stress resistance compared to Japanese cherry blossoms. It is suitable for planting in tropical or subtropical regions of southern China (Wang et al., 2024a, 2024b; Zou et al., 2013). Given its broad market application prospects, there is an urgent demand for large-scale seedling propagation.

However, conventional propagation methods for *C. campanulata* face notable limitations. Seed propagation often leads to trait segregation in offspring, while vegetative techniques such as cutting and grafting—although capable of preserving genetic integrity—are constrained by seasonal dependence, limited scion availability, and an under−optimized technical framework. These factors collectively hinder the standardization and large−scale production of uniform plantlets (Hu et al., 2019). Currently, the qualification rate for domestically produced *C. campanulata* seedlings remains as low as 58.2%, falling short of the ≥85% threshold required for industrial-scale production (State Forestry and Grassland Administration of China, 2021). Tissue culture has a high propagation coefficient and a short seedling formation cycle. It can maintain the stability of varieties and preserve their excellent characteristics, making it an effective approach for the large-scale seedling cultivation of cherry blossoms (Zhang and Jiang, 2016). As early as 1974, Boxus and Quoirin (1974) began to attempt to propagate cherry blossoms using tissue culture technology. Since then, researchers have continuously carried out research related to the tissue culture of cherry blossoms. However, existing studies have primarily focused on establishing basic regeneration systems, with objectives largely confined to small-scale laboratory cultivation. The medium formulations and culture conditions lack systematic optimization for industrial-scale application. Consequently, a standardized, reproducible technical system suitable for industrial propagation remains underdeveloped, particularly with respect to proliferation efficiency, rooting consistency, verification of genetic stability across multiple subcultures, and the integrated optimization of the complete workflow from inoculation to acclimatization.

The core requirements for industrial-scale micropropagation are high stability, high proliferation efficiency, standardized operation, and scalable integration with field production (Honda and Kobayashi, 2004; Abdalla et al., 2022). However, existing protocols are generally plagued by issues such as unstable proliferation coefficients, high vitrification rates, and low transplant survival rates, which fail to meet the industrial demand for reliable batch-output of elite plantlets. For instance, in the culture system established by Sanson et al. (2024), the rooting rate was only 63%, with an acclimatization period as long as 120 days. Chen et al. (2023) merely screened the culture media for the tissue culture stage of *C. campanulata* but did not involve acclimatization and transplantation, making direct industrial application difficult. Luo et al. (2021) developed a regeneration system for *Cerasus serrulata* var. *lannesiana* cv. ‘Grandiflora’, yet they did not verify its genetic stability; after multiple subcultures, leaf yellowing and increased mortality were observed. The market still lacks high-quality, genetically uniform standardized tissue-cultured plantlets, which severely restricts the large-scale application of this species in landscaping and related industries. Therefore, establishing an optimized, reproducible, and industrially applicable micropropagation system for *C. campanulata*, along with the promotion of high-quality tissue-cultured plantlets, is now crucial for overcoming this key industrial development bottleneck.

Therefore, the present study took *C. campanulata* as the experimental material, aiming to establish a highly efficient, stable, and reproducible micropropagation system toward industrial-scale production. It systematically optimized key culture factors for proliferation and rooting using an orthogonal experimental design. Beyond establishing an efficient regeneration protocol, it also verified the genetic stability of the regenerated plantlets over subculture generations and accomplished the complete cycle from *in vitro* culture to pot cultivation. This integrated approach provides a scalable and verified protocol, directly addressing the gap between laboratory research and the commercial need for standardized, high-quality plantlets.

## 2 Materials and methods

### 2.1 Plant material

Sterile tissue-cultured seedlings of *Cerasus campanulata* Maxim., derived from a pre-established regeneration system, were used to optimize the proliferation and rooting stages. The original explants for establishing this system were axillary bud-bearing stem segments collected from Fenghua, Ningbo, China.

Prior to sterilization, the segments were surface-wiped with 75% (v/v) ethanol, the outer layers of the buds were carefully peeled off, and the bud-stem junctions were trimmed smooth to ensure the bud apices remained tightly enclosed. The pretreated explants were then agitated in a detergent solution for 15 min and rinsed thoroughly under running water, followed by sequential surface sterilization in 75% (v/v) ethanol for 30 s and 1% (v/v) sodium hypochlorite (NaClO) for 5 min. After sterilization, the explants were rinsed 2–3 times with sterile distilled water. Finally, they were cultured on primary and adventitious bud induction media to generate sterile stock plantlets, which were maintained and subcultured for use in subsequent experiments.

### 2.2 Culture conditions

The culture medium contained 30 g/L sucrose and 7 g/L agar, adjusted to pH 5.8 ± 0.1, and autoclaved at 121 °C for 20 minutes. The cultivation conditions were as follows: temperature, (23 ± 2) °C; light intensity, 3,500–4,000 lx; and photoperiod, 12 h.

### 2.3 Proliferation culture

Single adventitious buds (approximately 2–3 cm in height) from tissue-cultured seedlings of *C. campanulata* were excised and inoculated onto the respective media. An orthogonal experiment was designed with four factors at three levels each: basal medium (½MS, MS, WPM), banana powder (BP; food-grade, Bozhou Huazhitang Biotechnology Co., Ltd.; derived from freeze-dried ripe bananas; same production batch used throughout the study, added directly to the medium before autoclaving to ensure consistency), concentration (0, 2.5, 5 g/L), NAA concentration (0, 0.1, 0.3 mg/L), and 6-BA concentration (0, 0.5, 2.0 mg/L). An L_9_(3^4^) orthogonal array was employed, resulting in a total of 9 treatment combinations (**Table 1**). 65 days after inoculation, the survival rate (the number of surviving buds/the number of inoculated buds), the adventitious bud induction rate (the number of proliferated buds/the number of inoculated buds), the proliferation coefficient (the total number of newly proliferated buds/the number of inoculated buds that exhibited proliferation) and the bud height were counted. Each treatment consisted of 10 culture bottles inoculated with 5 shoots each, with three independent replicates.

**Table 1:** The L_9_(3^4^) orthogonal design for proliferation culture.

Treatment	Basal Medium	BP (g/L)	NAA (mg/L)	6-BA (mg/L)
A1	½MS	0	0	0
A2	½MS	2.5	0.1	0.5
A3	½MS	5	0.3	2
A4	MS	0	0.1	2
A5	MS	2.5	0.3	0
A6	MS	5	0	0.5
A7	WPM	0	0.3	0.5
A8	WPM	2.5	0	2
A9	WPM	5	0.1	0

### 2.4 Rooting culture

Adventitious buds (3–4 cm in height) were excised and inoculated onto the respective media. An orthogonal experiment was designed with four factors at three levels each: basal medium (½MS, MS, WPM), BP concentration (0, 2.5, 5 g/L), activated carbon (AC) concentration (0, 0.2, 0.5 g/L), and plant growth regulator (PGR) type/concentration (0, 0.1 mg/L NAA, 0.1 mg/L IBA). An L_9_(3^4^) orthogonal array was employed, resulting in 9 treatment combinations (**Table 2**). Rooting rate (number of rooted buds/number of inoculated buds) was recorded every 5 days, starting from day 15. After 60 days, the survival rate, root number, and plant height were measured. Each treatment consisted of 30 buds and was replicated three times.

**Table 2:** The L_9_(3³) orthogonal design for rooting culture.

Treatment	Basal Medium	BP (g/L)	AC (g/L)	PGR (mg/L)
B1	½MS	0	0	0
B2	½MS	2.5	0.2	NAA 0.1
B3	½MS	5	0.5	IBA 0.1
B4	MS	0	0.2	IBA 0.1
B5	MS	2.5	0.5	0
B6	MS	5	0	NAA 0.1
B7	WPM	0	0.5	NAA 0.1
B8	WPM	2.5	0	IBA 0.1
B9	WPM	5	0.2	0

To further verify the results, adventitious buds were cultured for rooting on five distinct media(C1-C5): ½MS, ½MS + 0.5 g/L AC, ½MS + 0.05 mg/L IBA + 0.5 g/L AC, ½MS + 0.1 mg/L IBA + 0.5 g/L AC, and ½MS + 0.2 mg/L IBA + 0.5 g/L AC. The survival rate, rooting rate, and plant height were subsequently statistically analyzed.

### 2.5 Transplantation

The rooted plantlets were transferred to a greenhouse for acclimatization. They were removed from the culture vessel, and the medium adhering to the roots was gently washed off with water. The adventitious roots were then trimmed, leaving approximately 0.5 cm at the base. The plantlets were subsequently transplanted into a plug tray filled with substrate. During the first 15 days, the ambient humidity was maintained above 90%, after which the plantlets were cultivated under normal greenhouse conditions. The survival rate was calculated after 30 days. Subsequently, the acclimatized plug seedlings were potted in a 1:1 (v/v) mixture of peat and coconut coir. Plant height was measured every 20 days. After 120 days of pot cultivation, the leaf length, leaf width, and total leaf number were recorded. The experiment was conducted with three replicates, with five plantlets per replicate.

### 2.6 ISSR analysis of micropropagated plantlets

To assess genetic stability, DNA was extracted from three randomly selected tissue-cultured plantlets from each of the first three subculture generations. The extracted DNA was used as a template to screen 107 random primers (**Supplementary Table S1**). Four primers (**Table 3**) that produced clear and reproducible amplification profiles were selected. PCR amplification was then performed using the following reaction system and thermocycling program: the total volume of the ISSR-PCR reaction system was 20 μL, consisting of 1 μL primer, 1 μL template DNA, 10 μL Mix, and 8 μL ddH_2_O. The thermocycling program included an initial denaturation step at 95°C for 3 min, followed by 40 amplification cycles (each cycle comprised denaturation at 95°C for 30 s, annealing at 37°C for 30 s, and extension at 72°C for 1 min), a final extension step at 72°C for 6 min, and a hold step at 25°C indefinitely.

**Table 3:** The primers and their sequence are used for ISSR analysis.

Primers	Sequence (5’→3’)	Number of bands	Band size range (bp)
ISSR-892	TAGATCTGATATCTGAATTCCC	2	500-2 000
ISSR-873	GACAGACAGACAGACA	4	250-2 000
ISSR-M03	CACCACACACARG	3	250-2 000
ISSR-M08	AGCAGCAGCAGCAY	1	750-1 000

R = (A, G),Y = (C, T)

### 2.7 Data analysis

Statistical analysis was conducted using WPS Office and IBM SPSS Statistics 22.

## 3 Results

### 3.1 Effects of basic media, BP, and PGR concentrations on proliferation of *C. campanulata*

**Figure 1:**
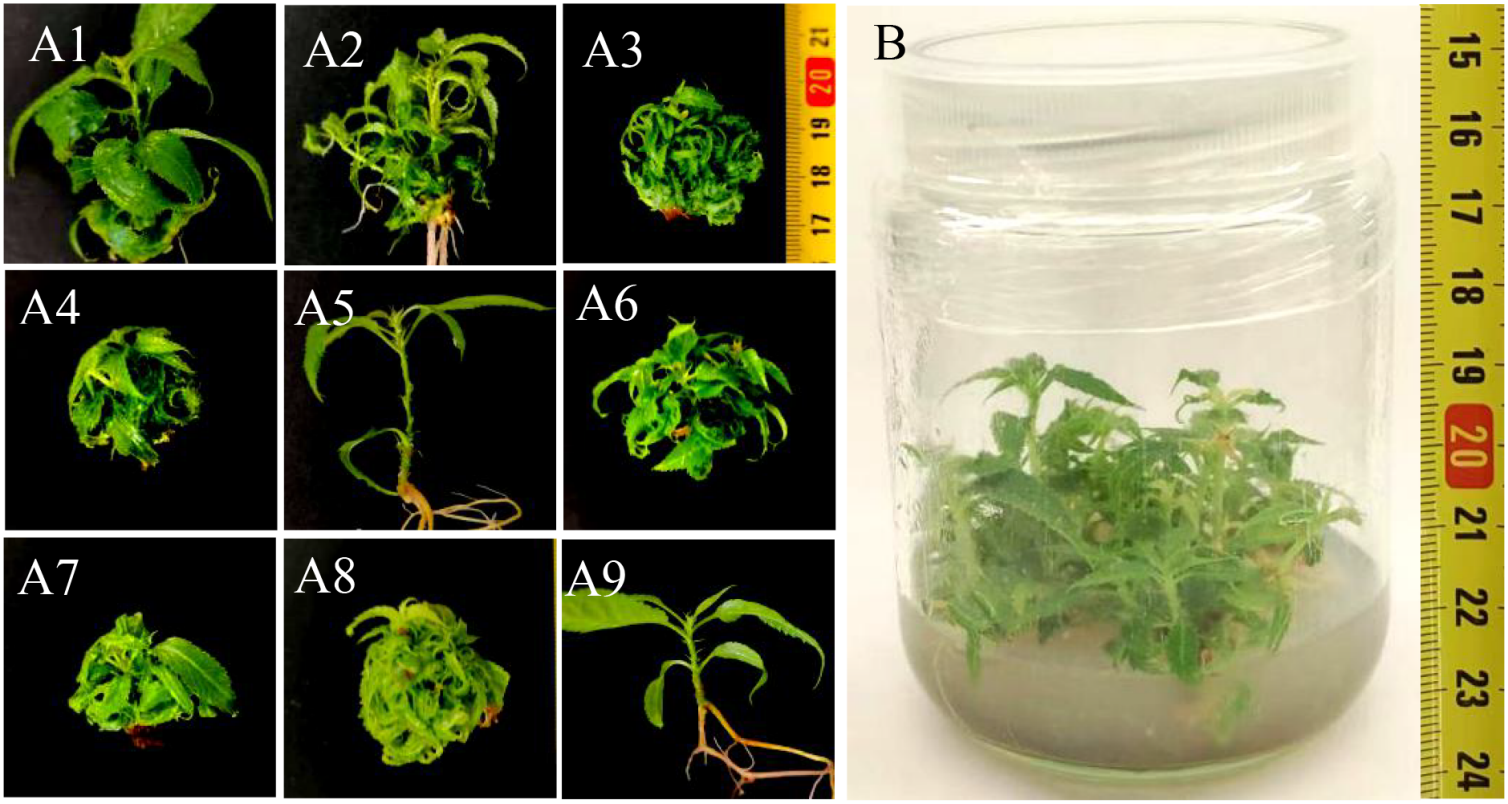
Effects of different medium formulations on the proliferation of *C. campanulata.* Growth status of adventitious bud clusters in treatment groups (A1–A9); **(B)** Proliferation Culture.

Based on the analysis of the growth status and range analysis (**Tables 4**, **5**; **Figure 1**), during the proliferation stage of *C. campanulata*, for the survival rate, the influencing order of factors is basal medium > 6-BA > NAA > BP. For the induction rate, the influencing order of factors is 6-BA > BP > basal medium > NAA. While for the proliferation coefficient, the influencing order is 6-BA > basal medium > BP > NAA. In terms of the bud height, the influencing order is 6-BA > basal medium > NAA > BP.

**Table 4:** Effects of basic media, BP, and PGR concentrations on proliferation of *C. campanulata*.

Treatment	Survival rate (%)	Proliferation rate (%)	Proliferation coefficient	Bud height (cm)	Bud seedling status
A1	93.33 ± 0.06 a	6.67 ± 0.12 c	0.33 ± 0.58 e	3.89 ± 0.15 b	vigorous, tall
A2	100.00 ± 0.00 a	96.67 ± 0.06 a	4.03 ± 0.16 b	4.57 ± 0.27 a	moderately vigorous, tall
A3	100.00 ± 0.00 a	100.00 ± 0.00 a	7.50 ± 0.20 a	2.84 ± 0.13 c	slender, leaf curling, dwarfed
A4	96.67 ± 0.06 a	86.67 ± 0.15 a	2.52 ± 0.20 cd	2.67 ± 0.12 c	slender, leaf curling, dwarfed
A5	80.00 ± 0.10 b	0.00 ± 0.00 c	0.00 ± 0.00 e	3.68 ± 0.04 b	vigorous, tall
A6	96.67 ± 0.06 a	90.00 ± 0.10 a	2.60 ± 0.34 c	2.39 ± 0.10 d	weak, dwarfed
A7	63.33 ± 0.06 c	56.67 ± 0.12 b	1.97 ± 0.37 d	2.19 ± 0.10 d	weak, leaf curling, dwarfed, chlorosis
A8	93.33 ± 0.06 a	90.00 ± 0.10 a	2.78 ± 0.10 c	2.28 ± 0.11 d	weak, leaf curling, dwarfed
A9	73.33 ± 0.12 bc	10.00 ± 0.17 c	0.33 ± 0.58 e	3.87 ± 0.12 b	vigorous, tall, chlorosis

Average value ± standard deviation. Values in the same column followed by different lowercase letters indicate significant difference (*P* < 0.05). Lowercase letters (a–e) are assigned in descending order of mean values within each column; values sharing the same letter are not significantly different.

**Table 5:** Range analysis of proliferation parameters.

Factor		Basal medium	BP	NAA	6-BA
Survival Rate	k1	0.978	0.844	0.944	0.822
	k2	0.911	0.911	0.900	0.867
	k3	0.767	0.900	0.811	0.967
	R	0.211	0.067	0.133	0.144
Proliferation Rate	k1	0.678	0.500	0.622	0.056
	k2	0.589	0.622	0.644	0.811
	k3	0.522	0.667	0.522	0.922
	R	0.156	0.167	0.122	0.867
Proliferation Coefficient	k1	3.954	1.607	1.904	0.222

Taking into consideration the four dependent variables (survival rate, induction rate, proliferation coefficient, and bud height), the optimal basal medium for *C. campanulata* tissue culture is ½MS, followed by MS, while WPM exhibits inferior performance. BP at concentrations of 2.5–5 g/L enhanced proliferation in *C. campanulata*. While promoting proliferation, elevated NAA concentrations reduced the survival rate of bud seedlings. A concentration of 0.1 mg/L was identified as optimal for achieving effective proliferation without significant survival loss. While higher 6-BA concentrations (up to 2.0 mg/L) improved adventitious bud survival and proliferation, they compromised morphological quality, producing stunted, thin, and curled shoots; additionally, the average bud height was significantly reduced by 37.86%. Therefore, 0.5 mg/L 6-BA was chosen to achieve effective propagation without suboptimal morphological effects.

A culture medium formulation of ½MS, 0.1 mg/L NAA, 0.5 mg/L 6-BA, and 2.5 g/L BP supported robust proliferation of *C. campanulata* that met production requirements, while also ensuring high survival rates and excellent plantlet morphology.

### 3.2 Effects of Basal Medium, BP, AC and PGR Concentrations on Rooting of *C. campanulata*

Evaluation of five dependent variables (survival rate, rooting rate, root number, plant height, and transplantation survival rate) from orthogonal experiments on rooting culture identified ½MS as the optimal basal medium (**Tables 6**, **7**). The addition of BP inhibited rooting, whereas both AC and PGR promoted it. Specifically, the inclusion of 0.5 g/L AC with either 0.1 mg/L NAA or 0.1 mg/L IBA significantly enhanced the rooting rate and stimulated shoot elongation. However, treatments with high rooting rates did not necessarily yield high transplant survival. The correlation between rooting rate and transplant survival rate was weak (correlation coefficient, r = 0.312), while the correlation between root number and transplant survival rate was even weaker (r = 0.160). Notably, plant height showed a weak negative correlation with transplant survival rate (r = -0.310).

**Table 6:** Effects of basic media, BA, AC and PGR concentrations on the rooting culture of *C. campanulata*.

Treatment	Survival rate (%)	Rooting rate (%)	Number of roots	Seedling height (cm)	Transplant survival rate (%)
B1	90.00 ± 0.06 a	88.73 ± 0.04 ab	5.51 ± 0.97 a	5.23 ± 0.10 b	88.10
B2	53.33 ± 0.06 c	89.26 ± 0.04 ab	4.79 ± 0.58 ab	5.15 ± 0.03 b	32.43
B3	63.33 ± 0.13 bc	90.90 ± 0.04 ab	5.21 ± 0.19 ab	5.50 ± 0.29 a	29.27
B4	85.56 ± 0.16 a	97.70 ± 0.04 a	5.03 ± 0.27 ab	5.56 ± 0.10 a	20.29
B5	46.67 ± 0.10 c	95.24 ± 0.08 ab	4.65 ± 0.23 b	5.19 ± 0.14 b	15.63
B6	42.22 ± 0.11 c	100.00 ± 0.00 a	5.10 ± 0.26 ab	4.83 ± 0.09 c	13.33
B7	87.78 ± 0.10 a	79.09 ± 0.16 b	3.54 ± 0.19 c	4.38 ± 0.08 d	36.73
B8	77.78 ± 0.17 ab	48.68 ± 0.14 c	3.07 ± 0.36 c	3.95 ± 0.19 e	40.63
B9	58.89 ± 0.07 bc	40.00 ± 0.14 c	1.97 ± 0.06 d	3.66 ± 0.13 f	68.29

**Table 7:** Range analysis of rootting parameters.

Factor		Basal medium	BP	AC	PGR
Survival Rate	k1	0.689	0.878	0.700	0.652
	k2	0.581	0.593	0.659	0.611
	k3	0.748	0.548	0.659	0.756
	R	0.167	0.330	0.041	0.144
Rooting rate	k1	0.896	0.885	0.791	0.747
	k2	0.976	0.777	0.757	0.895
	k3	0.559	0.770	0.884	0.791
	R	0.417	0.115	0.128	0.148
Number of Roots	k1	5.169	4.694	4.561	4.044
	k2	4.928	4.170	3.927	4.476
	k3	2.860	4.093	4.469	4.438
	R	2.309	0.601	0.633	0.432
Seedling height	k1	5.294	5.057	4.669	4.695
	k2	5.194	4.764	4.794	4.787
	k3	3.997	4.664	5.023	5.004
	R	1.297	0.394	0.354	0.309
Transplant survival rate	k1	0.499	0.484	0.474	0.573
	k2	0.164	0.296	0.403	0.275
	k3	0.486	0.370	0.272	0.301
	R	0.335	0.188	0.201	0.298

**Figure 2:**
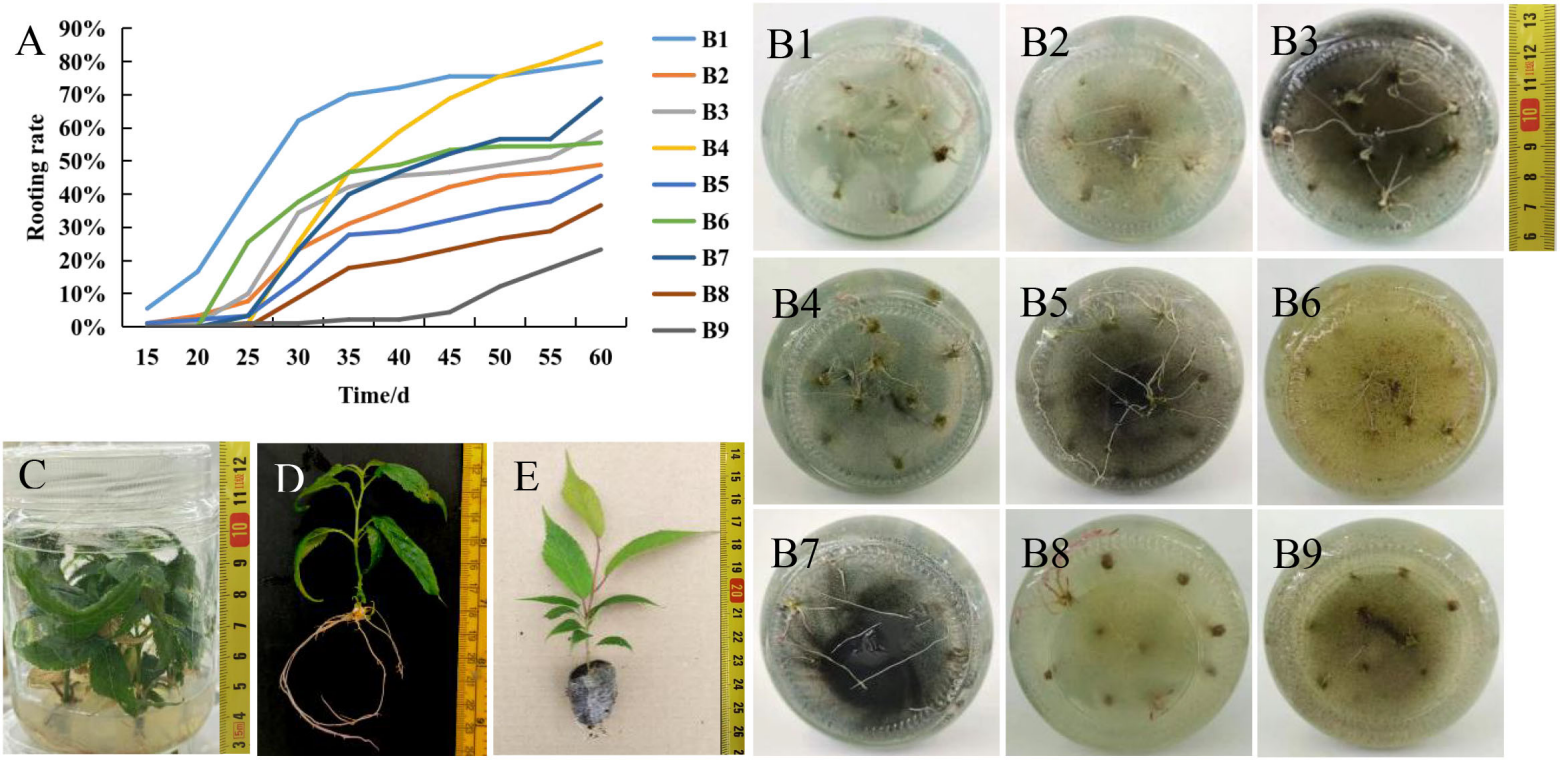
Effects of different medium formulations on the rooting of *C. campanulata.***(A)** Rooting rate of *C. campanulata* recorded at 5-day intervals in different culture media formulations. **(B)** Rooting status in B1-B9 medium; **(C)** Rooting culture; **(D)** Rooting seedlings; **(E)** Plug seedling.

The B1 treatment (½MS basal medium) induced roots with uniform morphology, sufficient thickness, and moderate length, resulting in high transplant survival. In contrast, treatments B2–B6, supplemented with exogenous substances (e.g., BP, AC, PGRs), exhibited high rooting rates but low transplant survival. This may be attributed to hormonal and nutritional imbalances after transplantation, leading to poor environmental adaptation and eventual mortality of the transplanted seedlings. Therefore, transplant survival is more closely related to the morphological robustness of the seedlings and root quality (such as uniformity and thickness) rather than merely the rooting rate or root number. Additionally, in the B1 treatment group, rooting initiated most rapidly (**Figure 2**): roots emerged sequentially within two weeks and the rooting process stabilized by 45 days. At this stage, the rooting performance reached a relatively optimal level, with a rooting rate exceeding 88%, an average root number per plant exceeding five, and an average plant height of 5.23 cm—conditions suitable for transplantation, achieving a transplant survival rate of over 88%. After further verification, the results also indicated that the basal medium ½MS could meet the production requirements. Thus, ½MS was identified as the optimal medium for the rooting stage of *C. campanulata*.

### 3.3 Growth of *C. campanulata* tissue-cultured seedlings in pots

**Figure 3:**
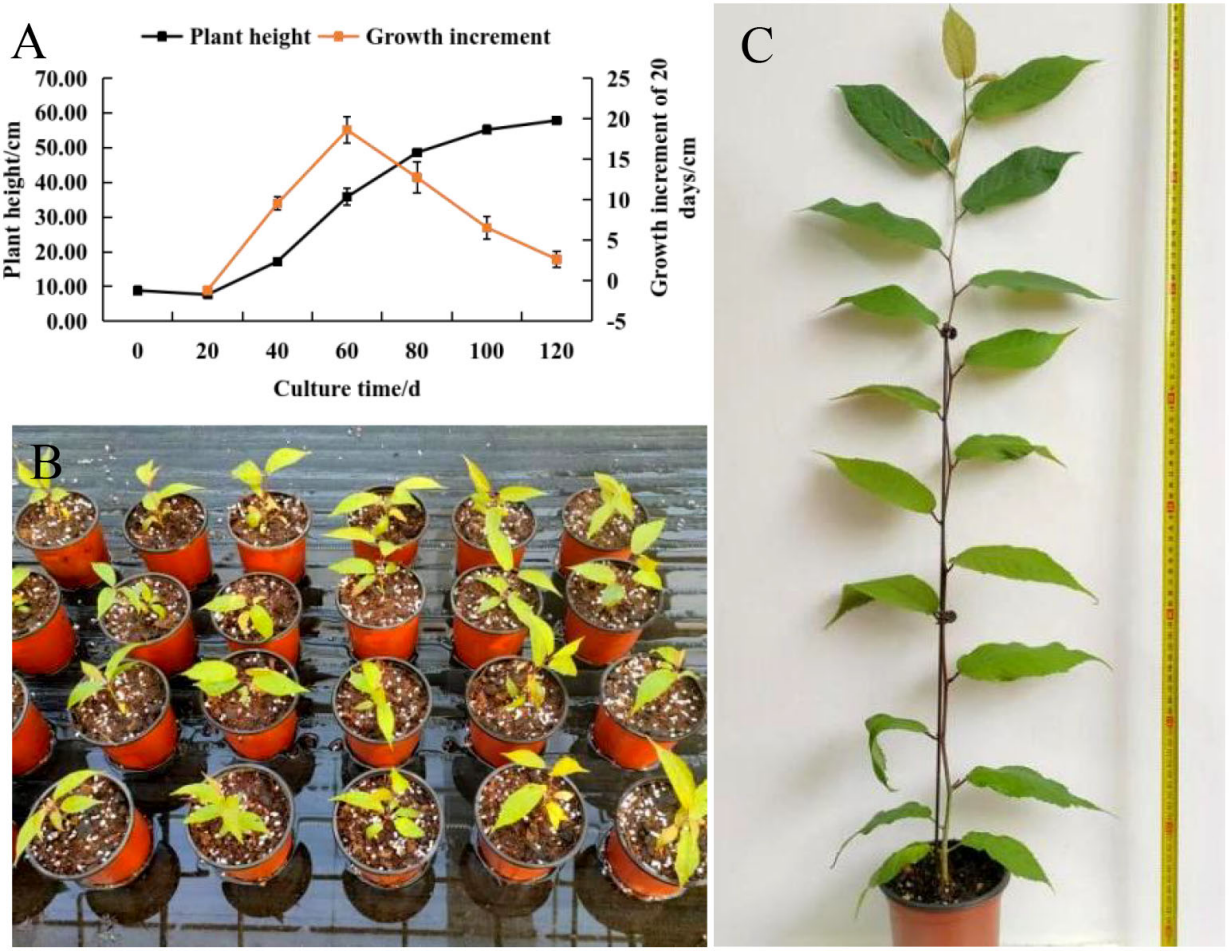
The growth status of *C. campanulata* in pots. **(A)** Record of the plant height and growth increment of *C. campanulata*; **(B)** Initial state of *C. campanulata* planting; **(C)** State of *C. campanulata* 120 days after planting.

As shown in **Figure 3-A**, *C. campanulata* seedlings exhibited rapid growth between 20 and 100 days after transplantation into pots. The peak increase in plant height occurred between 40 and 60 days, with an average growth of 18.61 cm during this 20-day period. The growth rate slowed after 100 days and had largely plateaued by 120 days, at which point the plants had reached an average height of 57.83 cm, with leaf length of 15.03 cm, leaf width of 6.31 cm, and a total of 17 leaves. These morphological metrics indicate that the plants conformed to the Grade-1 container seedling standards for *C. campanulata* (State Forestry and Grassland Administration of China, 2021).

### 3.4 Assessment of Genetic Stability in Micropropagated Plantlets by ISSR analysis

**Figure 4:**
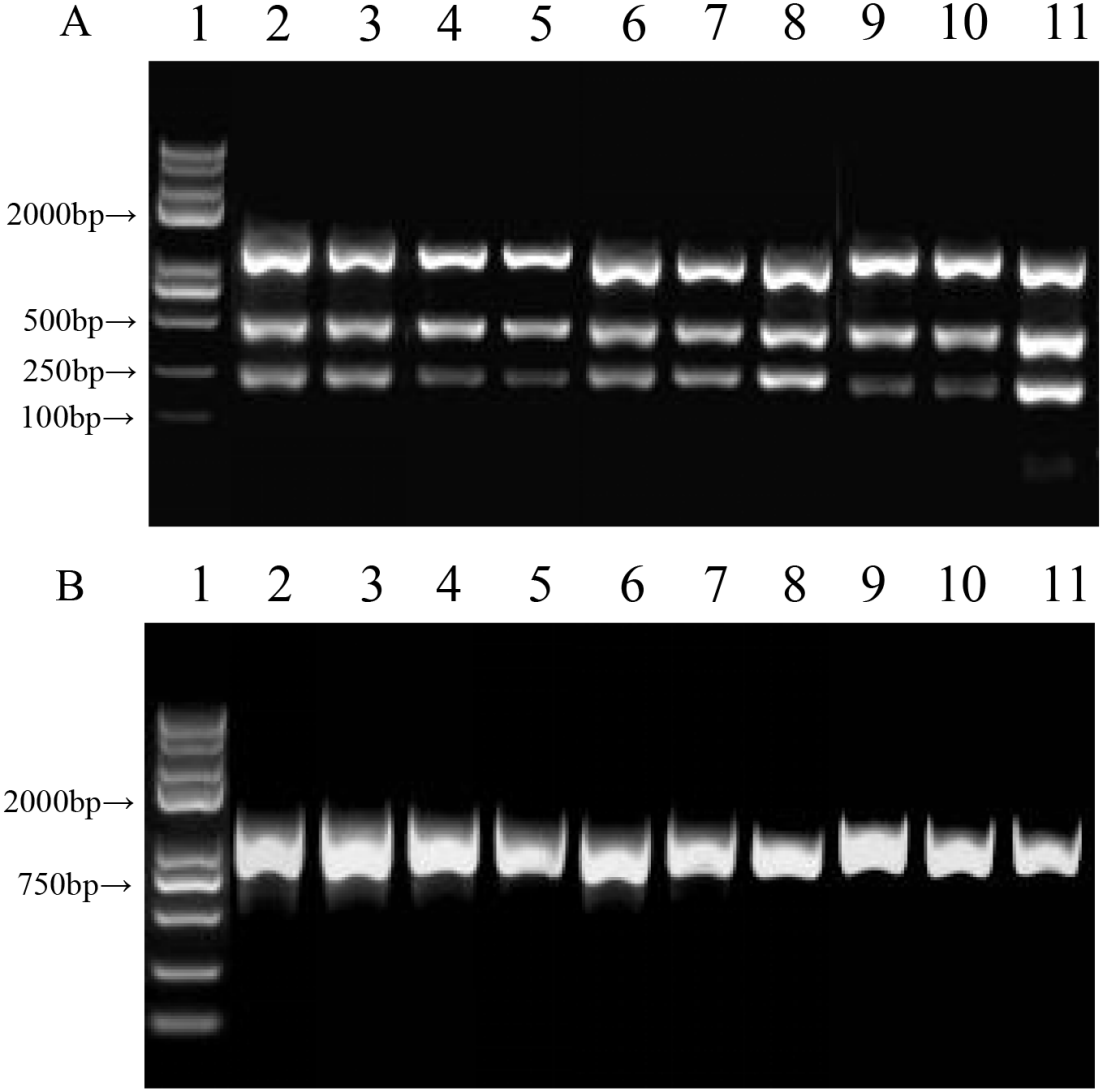
Genetic fidelity analysis of *in vitro* micropropagated *C. campanulata* plantlets across three successive subculture generations. **(A)** Amplification bands generated by primer ISSR-M03; **(B)** Amplification bands generated by primer ISSR-M08; Lane 1: DNA molecular weight marker; Lane 2: Mother plant (control); Lanes 3–5: Three independently selected plantlets from the first subculture generation (representing three biological replicates); Lanes 6–8: Three independently selected plantlets from the second subculture generation; Lanes 9–11: Three independently selected plantlets from the third subculture generation.

After screening the aforementioned 107 ISSR primers, four primers (ISSR-892, ISSR-873, ISSR-M03, and ISSR-M08) exhibiting clear and reproducible banding patterns were selected. These primers were used to amplify genomic DNA from the mother plant and from nine randomly selected micropropagated plantlets (three per generation across three generations). Of the selected primers, ISSR-892 amplified two distinct bands, ISSR-873 produced four clear bands, ISSR-M03 generated three well-resolved bands, and ISSR-M08 yielded one sharp band (**Figure 4**). In toal, 10 clear and scorable loci were amplified, with fragment sizes ranging from 250 bp to 2–000 bp (see **Table 3** for details per primer). When the same primers were used, the amplification profiles were consistent across the mother plant and all three generations of micropropagated plantlets, with no polymorphic bands detected. This indicated that no observable genetic variations had occurred within the detected genomic regions following three subculture cycles.

## 4 Discussion

This study systematically evaluated the effects of basal medium and 6-BA on the *in vitro* proliferation of *C. campanulata*. The results showed that among the three basal media tested—½MS, MS, and WPM—½MS yielded the best proliferation outcome, outperforming both MS and WPM. This ranking inversely correlated with their ammonium nitrate content (MS > ½MS > WPM), indicating that the superior performance of ½MS is closely associated with its moderate ammonium nitrate concentration. This finding not only aligns with the view of Chen et al. (2011) that reducing ammonium nitrogen can mitigate vitrification but further suggests the existence of an “optimal range” for nitrogen supply, rather than a simple “lower is better” approach. This differs from the results of Chen et al. (2023), who reported that WPM was more suitable for the micropropagation of *Cerasus campanulata* var. wuyiensis, possibly reflecting interspecific or genotypic differences in nutritional requirements.Regarding hormonal regulation, 6-BA significantly promoted proliferation, consistent with findings from most tissue culture studies on cherry blossoms (Li et al., 2014, 2020; Wang, 2021). However, this study further revealed that excessively high concentrations of 6-BA (e.g., 1.0 mg/L) led to thin shoots and leaf curling—symptoms often attributed in previous studies to high salt-induced phenolic oxidation in the basal medium (Zhang and Jiang, 2015). By comparing different treatments, we propose that in the proliferation culture of *C. campanulata*, morphological abnormalities are primarily associated with cytokinin overload, while the influence of salt stress may be partially masked after medium selection. Therefore, this study identifies 0.5 mg/L as the suitable concentration of 6-BA, promoting proliferation while maintaining robust shoot growth. Additionally, supplementation with low concentrations of NAA (0.1 mg/L) and BP (2.5–5 g/L) exhibited a synergistic promotive effect on proliferation. This suggests that, beyond optimizing the basal medium and cytokinin, appropriate organic additives and auxin can further enhance culture efficiency, potentially through mechanisms involving carbon source supplementation and the modulation of endogenous hormone networks (Zhang and Jiang, 2016).

Studies have shown that the rooting culture of cherry blossoms typically uses ½MS as the basal medium (Li et al., 2020; Wang, 2021; Liu et al., 2020; Duţă, 2012), while some studies employ modified MS or WPM media (Huang et al., 2002; Sanson et al., 2024). Notably, distinct strategies exist across studies regarding auxin application: for example, Lv et al. (2006) supplemented ½MS with 1.0 mg/L NAA and 0.2 mg/L IBA during the rooting stage of *C. campanulata*, significantly enhancing rooting efficiency; whereas Zhang and Jiang (2015) suggested that a short-term high-concentration auxin treatment followed by subculture onto hormone-free medium can avoid sustained inhibition of rooting. These differences indicate that the effect of auxins may depend not only on concentration but also on the timing of treatment and the overall balance of the culture system. In this study, ½MS was identified as the optimal basal medium, and the addition of 0.5 g/L AC along with 0.1 mg/L NAA or IBA helped further improve the rooting rate and promote shoot elongation. However, in the treatment where only ½MS was used without any exogenous auxin, rooting initiated most rapidly, and both the final rooting performance and transplant survival rate were excellent, indicating that ½MS alone can meet the requirements for efficient rooting in this system. This finding aligns with results reported by Luo et al. (2021) for *C. serrulata* ‘Grandiflora’, whose plantlets achieved a 100% rooting rate on ½MS basal medium, while the addition of NAA or IBA inhibited rhizogenesis. We speculate that as the culture period extends, plant hormones may accumulate dynamically, leading to changes in the plant’s hormonal response thresholds and reducing the demand for exogenous regulators. Alternatively, the plants may have developed hormone-autotrophic adaptation (i.e., activation of endogenous hormone synthesis pathways), thereby acquiring regenerative capacity independent of exogenous PGRs (George et al., 2008). In summary, whether exogenous auxin is added is not the sole determinant of successful rooting in cherry species. Its effects may vary depending on genotype, basal medium composition, culture stage, and endogenous hormone levels.

While the consistent ISSR amplification profiles across three subculture generations indicate genetic stability, the limitations of the chosen methodology must be acknowledged. One key constraint is that, as dominant markers, ISSRs cannot distinguish between homozygous and heterozygous states. Furthermore, they sample only specific repetitive regions of the genome, offering a partial view rather than a comprehensive genomic scan (Debnath, 2012). Despite these well-recognized methodological limitations, ISSR analysis remains a widely validated and efficient tool for the initial screening of somaclonal variation in micropropagated plants. This includes its successful application in the rapid propagation of woody Rosaceae species, such as cherry (Prunus pseudocerasus), to verify genetic integrity (Zhang et al., 2023), as well as in the conservation of other plant species (Al-Qurainy et al., 2018). The complete absence of polymorphic bands across all ten loci in this study provides robust evidence for genetic fidelity within the regions surveyed under our culture conditions. An additional consideration is that the assessment was confined to three subculture cycles. This scope aligns with the validation phase of micropropagation protocols, where stability through early generations (typically 3rd to 5th) is considered critical, as these often serve as the “core mother stock” for production scaling (Hou et al., 2022). It should be noted, however, that this does not preclude the possibility of variation emerging in later, extended subcultures beyond the tested range. To address these limitations and further strengthen the conclusions for large-scale application, future work could: (1) employ a broader set of molecular markers (e.g., SSRs, SNPs) for more genome-wide coverage; (2) extend stability monitoring to later subculture generations (e.g., 5-10); and (3) integrate phenotypic, physiological, and cytological assessments with molecular data. Such a multidimensional approach would provide a more comprehensive validation of the long-term genetic stability of *C. campanulata* in commercial micropropagation systems.

In summary, an efficient micropropagation system for *Cerasus campanulata* was established in this study. The optimal proliferation medium was ½MS supplemented with 0.1 mg/L NAA, 0.5 mg/L 6-BA, and 2.5 g/L banana powder, yielding a survival rate of 100.00%, an induction rate of 96.67%, a proliferation coefficient of 4.03, and well-developed shoots averaging 4.57 cm in height. For rooting, ½MS basal medium alone achieved a rooting rate and transplant survival rate both exceeding 88%, with an average of 5 roots per plantlet. Regenerated plants acclimatized successfully and exhibited vigorous growth in subsequent pot cultivation. ISSR marker analysis across three subculture cycles showed no obvious genetic variation in the regenerated plantlets. This protocol has been successfully implemented for pilot-scale seedling production, demonstrating its practical applicability. Overall, this system provides a reproducible and effective platform for the propagation of *C. campanulata*, supporting both further physiological studies and potential horticultural production.

## Data Availability

The datasets presented in this study can be found in online repositories. The names of the repository/repositories and accession number(s) can be found in the article/**Supplementary Material**.
