# Supplementary material for: Establishment of an industrialized micropropagation system for Cerasus campanulata Maxim
Source: Front Plant Sci. 2026 Feb 18;17:1766373. doi: 10.3389/fpls.2026.1766373 (PMC12957277; doi:10.3389/fpls.2026.1766373)
Supplement: Supplementary file 1 [file Table1.docx]

Supplementary Material

# Supplementary Tables

**Table S1.** The arbitrary primers and their Sequence are used for ISSR analysis

| Primers | Sequence (5'→3’) | Primers | Sequence (5'→3’) |
| --- | --- | --- | --- |
| ISSR-M01 | CACACACACACAR | ISSR-848 | CACACACACACACACARG |
| ISSR-M02 | CACCACACACARY | ISSR-849 | GTGTGTGTGTGTGTGTYA |
| ISSR-M03 | CACCACACACARG | ISSR-850 | GTGTGTGTGTGTGTGTYC |
| ISSR-M04 | GTGTGTGTGTGTYR | ISSR-851 | GTGTGTGTGTGTGTGTYG |
| ISSR-M05 | GCTGCTGCTGCTY | ISSR-852 | TCTCTCTCTCTCTCTCRA |
| ISSR-M06 | AGCAGCAGCAGCY | ISSR-853 | TCTCTCTCTCTCTCTCRT |
| ISSR-M08 | AGCAGCAGCAGCAY | ISSR-854 | TCTCTCTCTCTCTCTCRG |
| ISSR-801 | ATATATATATATATATT | 1SSR-855 | ACACACACACACACACYT |
| ISSR-802 | ATATATATATATATATG | ISSR-856 | ACACACACACACACACYA |
| ISSR-803 | ATATATATATATATATC | ISSR-857 | ACACACACACACACACYG |
| ISSR-804 | TATATATATATATATAA | ISSR-858 | TGTGTGTGTGTGTGTGRT |
| ISSR-805 | TATATATATATATATAC | ISSR-859 | TGTGTGTGTGTGTGTGRC |
| ISSR-806 | TATATATATATATATAG | ISSR-860 | TGTGTGTGTGTGTGTGRA |
| ISSR-807 | AGAGAGAGAGAGAGAGT | ISSR-861 | ACCACCACCACCACCACC |
| ISSR-808 | AGAGAGAGAGAGAGAGC | ISSR-862 | AGCAGCAGCAGCAGCAGC |
| ISSR-809 | AGAGAGAGAGAGAGAGG | ISSR-863 | AGTAGTAGTAGTAGTAGT |
| ISSR-810 | GAGAGAGAGAGAGAGAT | ISSR-864 | ATGATGATGATGATGATG |
| ISSR-811 | GAGAGAGAGAGAGAGAC | ISSR-865 | CCGCCGCCGCCGCCGCCG |
| ISSR-812 | GAGAGAGAGAGAGAGAA | ISSR-866 | CTCCTCCTCCTCCTCCTC |
| ISSR-813 | CTCTCTCTCTCTCTCTT | ISSR-867 | GGCGGCGGCGGCGGCGGC |
| ISSR-814 | CTCTCTCTCTCTCTCTA | ISSR-868 | GAAGAAGAAGAAGAAGAA |
| ISSR-815 | CTCTCTCTCTCTCTCTG | ISSR-869 | GTTGTTGTTGTTGTTGTT |
| ISSR-816 | CACACACACACACACAT | ISSR-870 | TGCTGCTGCTGCTGCTGC |
| ISSR-817 | CACACACACACACACAA | ISSR-871 | TATTATTATTATTATTAT |
| ISSR-818 | CACACACACACACACAG | ISSR-872 | GATAGATAGATAGATA |
| ISSR-819 | GTGTGTGTGTGTGTGTA | ISSR-873 | GACAGACAGACAGACA |
| ISSR-820 | GTGTGTGTGTGTGTGTC | ISSR-874 | CCCTCCCTCCCTCCCT |
| ISSR-821 | GTGTGTGIGTGIGTGTT | ISSR-875 | CTAGCTAGCTAGCTAG |
| ISSR-822 | TCTCTCTCTCTCTCTCA | ISSR-876 | GATAGATAGACAGACA |
| ISSR-823 | TCTCTCTCTCICTCTCC | ISSR-877 | TGCATGCATGCATGCA |
| ISSR-824 | TCTCTCTCTCTCTCTCG | ISSR-878 | GGATGGATGGATGGAT |
| ISSR-825 | ACACACACACACACACT | ISSR-879 | CTTCACTTCACTTCA |
| ISSR-826 | ACACACACACACACACC | ISSR-880 | GGAGAGGAGAGGAGA |
| ISSR-827 | ACACACACACACACACG | ISSR-881 | GGGTGGGGTGGGGTG |
| ISSR-828 | TGTGTGTGTGTGTGTGA | ISSR-882 | VBVATATATATATATAT |
| ISSR-829 | TGTGTGTGTGTGTGTGC | ISSR-883 | BVBTATATATATATATA |
| ISSR-830 | TGTGTGTGTGTGTGTGG | ISSR-884 | HBHAGAGAGAGAGAGAG |
| ISSR-831 | ATATATATATATATATYA | ISSR-885 | BHBGAGAGAGAGAGAGA |
| ISSR-832 | ATATATATATATATATYC | ISSR-886 | VDVCTCTCTCTCTCTCT |
| ISSR-833 | ATATATATATATATATYG | ISSR-887 | DVDTCTCTCTCTCTCTC |
| ISSR-834 | AGAGAGAGAGAGAGAGYT | ISSR-888 | BDBCACACACACACACA |
| ISSR-835 | AGAGAGAGAGAGAGAGYC | ISSR-889 | DBDACACACACACACAC |
| ISSR-836 | AGAGAGAGAGAGAGAGYA | ISSR-890 | VHVGTGTGTGTGTGTGT |
| ISSR-837 | TATATATATATATATART | ISSR-891 | HVHTGTGTGTGTGTGTG |
| ISSR-838 | TATATATATATATATARC | ISSR-892 | TAGATCTGATATCTGAATTCCC |
| ISSR-839 | TATATATATATATATARG | ISSR-893 | NNNNNNNNNNNNNNN |
| ISSR-840 | GAGAGAGAGAGAGAGAYT | ISSR-894 | TGGTAGCTCTTGATCANNNNN |
| ISSR-841 | GAGAGAGAGAGAGAGAYC | ISSR-895 | AGAGTTGGTAGCTCTTGATC |
| ISSR-842 | GAGAGAGAGAGAGAGAYG | ISSR-896 | AGGTCGCGGCCGCNNNNNNATG |
| ISSR-843 | CTCTCTCTCTCTCTCTRA | ISSR-897 | CCGACTCGAGNNNNNNATGTGG |
| ISSR-844 | CTCTCTCTCTCTCTCTRC | ISSR-898 | GATCAAGCTTNNNNNNATGTGG |
| ISSR-845 | CTCTCTCTCTCTCTCTRG | ISSR-899 | CATGGTGTTGGTCATTGTTCCA |
| ISSR-846 | CACACACACACACACART | ISSR-900 | ACTTCCCCACAGGTTAACACA |
| ISSR-847 | CACACACACACACACARC |  |  |

R = (A , G) ,Y = (C , T), V = (A , C , G) ( I. e. not T), B = (C , G, T) ( I. e. not A), H = (A , C , T) ( I. e. not G), D = (A , G, T) ( I. e. not C), N = (A , G, C , T)
